# Three New Species and a New Record of the Lichen Genus *Peltula* (Peltulaceae) from Helan Mountain in China

**DOI:** 10.3390/biology13080590

**Published:** 2024-08-05

**Authors:** Siying Wang, Gege Zhao, Dongling Niu, Liang Wang, Xia Ren, Jinai Wu, Hongbin Qu

**Affiliations:** 1 School of Life Sciences, Ningxia University, Yinchuan 750021, China; 12022131043@stu.nxu.edu.cn (S.W.); zgg17856919388@163.com (G.Z.); 2 Inner Mongolia Helan Mountain National Nature Reserve Administration, Alxa East County 750300, China; 13804736791@163.com (L.W.); 13948023111@163.com (X.R.); hlsgljxx@163.com (J.W.); 18648300678@163.com (H.Q.)

**Keywords:** lichen, Peltulaceae, systematic taxonomy, biodiversity, desert steppe region, Helan Mountain, species key

## Abstract

**Simple Summary:**

This paper reports on three new species and a new record of the lichen genus *Peltula* Nyl., highlighting research progress on lichen diversity in Helan Mountain. In this study, species were identified through a combination of morpho-anatomy, molecular systematics, and chemical substance detection. The results of the study enrich the data of *Peltula* and the study of lichens in Helan Mountain.

**Abstract:**

In this study, a systematic taxonomic analysis was carried out on the lichen genus *Peltula*, collected from Helan Mountain in China; three new species (*Peltula helanense*, *P. overlappine*, and *P. reticulata*) and a new record (*P. crispatula* (Nyl.) Egea) for China were identified. Four species were identified by morph-anatomical, chemical, and phylogenetic analyses by combining two gene loci (ITS and LSU). *Peltula helanense* is with tiny individual thalli up to 1mm, attached by creamy-white cylindrical rhizoids and apothecia filling the whole squamule. *Peltula overlappine* is characterized by thallus squamulose forming rosette-shaped patches and squamules with distinctive thickened margins. *Peltula reticulata* is characterized by brownish brown thallus and squamules with densely reticulate upper surface. *P. crispatula* is characterized by irregular squamules attached to a tuft of hyphae. The four species are described in detail, compared, and discussed with similar species, and images of morpho-anatomical structures of the four species are also provided. Moreover, a key to the species of *Peltula* from Helan Mountain is provided. The results enrich the data of the genus *Peltula* and also indicate that the rich diversity of lichen species in Helan Mountain is worthy of in-depth study.

## 1. Introduction

The lichen genus *Peltula* (Peltulaceae, Lichinales, Lichinomycetes, Ascomycota) was established by Nylander [1], with *Peltula radicata* Nyl. as the type species. After the establishment of *Peltula*, the genus was not further used for a long time, and many species now belonging to *Peltula* Nyl. had been included in *Heppia* Naeg. Until Gyelnik’s [2] revision of Heppiaceae, *Peltula* was used as a separate genus again. Later, Peltulaceae was established by Büdel [3], based on the type of ascocarps development, ascus characteristics, etc., and Peltula was moved into this family. According to the recent revision of the Peltulaceae by Frank et al. [4], Peltulaceae is verified as a monophyletic family through phylogenetic analyses, and *Phyllopeltula* and *Neoheppia* were moved into *Peltula*. At this point, the family Peltulaceae consists of only one genus *Peltula*. A total of 68 species of *Peltula* have been reported worldwide (https://indexfungorum.org/, accessed on 8 June 2024).

Species of *Peltula* have six growth types of thalli (peltate–umbilicate, squamulose-compound, squamulose–semifruticose, subfoliose-compound, crustose, and crustose–areolate) and ascus with lacerated gelatinous sheath; ascospores are single-celled, colorless, and transparent, with at least 16 or more than 100 [4,5,6]. Except for *P. langei*, Büdel and Elix contain the yellow pigments, Myeloconone D1 and D2 [7]; *P. africana* (Jatta) Swinscow and Krog and *P. impressa* (Vain.) Swinscow and Krog contain homosekikaic acid and unknown fatty acid [8]; few or no chemicals were detected in the other species.

*Peltula* is widespread worldwide, mainly in arid and semi-arid regions or in arid microenvironments of humid areas [3]. A total of 32 species have been reported in China [8,9,10,11], mainly distributed in Hong Kong [12,13], Taiwan [14], Gansu [11,15,16], Inner Mongolia [17], Beijing [10,11], and Ningxia [8,11]. Among them, Beijing currently has the most extensive distribution of *Peltula* in China, with a total of nine species reported.

Helan Mountain is a concentrated distribution area of endemic plants in the arid de-sert area in the middle of the Asian continent. With rich plant species and complex flora, it is a rare treasure trove of biological resources and an important center of biodiversity evolution in the arid region of northwestern China [18]. *Peltula* is a part of the lichen resources of Helan Mountain, mainly distributed in the premontane desert grassland area. It plays a vital role in stabilizing the land surface in the desert grassland area. In this study, we conducted a systematic taxonomic study of the genus *Peltula* collected from Helan Mountain. Eleven species were identified, including three new species (*Peltula helanense* S.Y. Wang and D.L. Niu, *P. overlappine* S.Y. Wang and D.L. Niu, and *P. reticulata* S.Y. Wang and D.L. Niu) and a new record (*P. crispatula* (Nyl.) Egea) from China.

## 2. Materials and Methods

### 2.1. Taxon Sampling

All specimens in this study were collected from Helan Mountain of China and preserved in NXAC (Botanical Herbarium, College of Life Sciences, Ningxia University, Yinchuan, China). The external morphology of the thallus and reproductive structures was observed, measured, and photographed using a stereo microscope (Soptop SZX12, Sunny Optical Technoligy (group) Co., Ltd., Ningbo, China); the anatomical structures were observed, measured, photographed, and recorded under electron microscope (Soptop RX50, Sunny Optical Technoligy (group) Co., Ltd., Ningbo, China). Microstructural preparations of thallus and reproductive structures were obtained using a freezing microtome (LEICA CM1950, Leica Holding B.V., Wetzlar, Germany). Some samples were sliced freehand by wetting the sample with distilled water and then cutting the thallus with a razor blade. A chemical color reaction was performed using 10% KOH aqueous solution, Ca(ClO)_2_ saturated aqueous solution, and 10% Lugol’s iodine solution. Lichen secondary metabolites were analyzed using TLC in system C (toluene: acetic acid = 170:30) [19].

### 2.2. DNA Extraction, Amplification, and Sequencing

Seven fresh specimens were selected for DNA extraction. ITS and LSU sequences were amplified by PCR. Genomic DNA was extracted using the Biospin Plant Genomic DNA Extraction Kit (Bruker Biospin Corporation, Etlingen, Germany). ITS was amplified using the primers ITS1F [20] and ITS4 [21,22]; LSU was amplified using the primers LR0R and LR7 [4]. The PCR mixture (25 μL) contained 25 μg of BSA, 1 U Taq DNA polymerase, dNTP (0.2 mmol/L), primers (0.5 μmol/L each), and PCR buffer, which was replenished to 25 μL with H_2_O. PCR cycling parameters consisted of the initial denaturation at 94 °C for 2 min, followed by denaturation at 94 °C for 30 s, annealing at 55 °C for 30 s, extension at 72 °C for 70 s, and finally, 72 °C extension for 2 min, and the amplification products were stored at 4 °C. The products were purified, identified, and sequenced using an ABI3730XL automated sequencer. The new sequences generated in this study were deposited in GenBank (https://www.ncbi.nlm.nih.gov/genbank/, accessed on 12 March 2024).

### 2.3. Sequence Alignment and Phylogenetic Analysis

Based on bidirectional sequencing results, sequence splicing was performed using Sequencher 4.1.4 with manual correction. In total, 120 reference sequences were downloaded from NCBI (https://www.ncbi.nlm.nih.gov/genbank/, accessed on 12 March 2024) and phylogenetically analyzed with the 15 sequences in this study (Table 1), and *Peccania* sp. and *Peccania terricola* were selected as outgroups. In PhyloSuite [23], sequence alignment was performed using MAFFT V 7 for ITS and LSU sequences separately [24], and Gblocks were used to eliminate uncertain regions [25]. Sequences were concatenated using the software’s concatenate function. The nucleotide substitution model before tree building was selected using ModelFinder [26]. The GTR+F+I+G4 model was chosen in Bayesian analyses and repeated for 700,000 generations, sampling every 1000 steps to construct the MrBayes phylogenetic tree [27]. The tree was aligned and beautified in iTOL (https://itol.embl.de/, accessed on 25 March 2024).

## 3. Results

### 3.1. Phylogenetic Analysis

**Figure 1:**
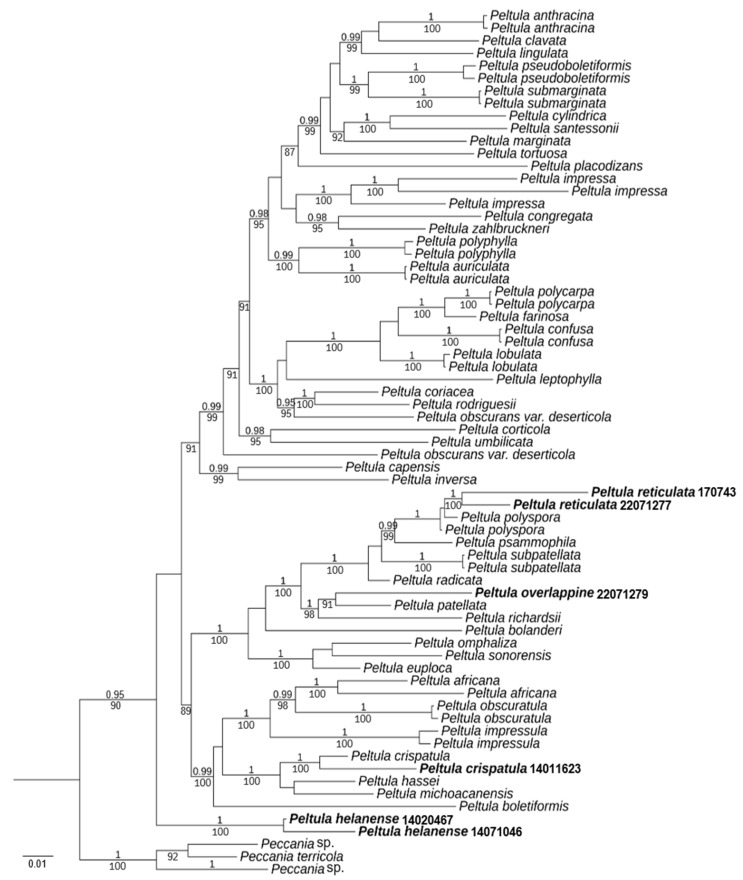
Phylogram based on ITS and LSU sequences of *Peltula* species; the MrBayes tree is shown with posterior probability (PP) values and bootstrap (BS) values of BI analysis. PP ≥ 0.95 and BS values ≥ 85 are plotted on the branches of the tree. PP values are indicated above branches, and BS values are below branches. The specimens of this study are indicated in bold. Branch lengths are scaled to the expected number of substitutions per site.

A total of 135 DNA sequences were used, including 15 new sequences (6 ITS, 9 LSU) (Table 1). In total, a data matrix corresponding to the ITS and LSU regions was generated with 1650 characters (ITS, 1-359; LSU, 360-1650). Bayesian Inference (BI) phylogenetic trees of the concatenated data set of the two gene markers were constructed (Figure 1).

The resulting tree showed two well-supported branches (PP = 1, BS = 100) corresponding to the new species *Peltula helanense* and the new record species *P. crispatula*. Moreover, almost all species had strong support. *P. reticulata* and *P. polyspora* are well-supported (PP = 1), but it is easy to distinguish them by their appearances. *P. overlappine* and *P. patellata* are also well-supported (BS = 91). However, the morphology and chemistry of the two species were distinct. Although the new species *P. helanense* is at the outermost part of the taxon, it is morphologically fully characterized as a species of *Peltula*. It has small squamules with ascus with lacerated gelatinous sheath, and ascospores are single-celled, colorless, and transparent.

### 3.2. TLC Results

**Figure 2:**
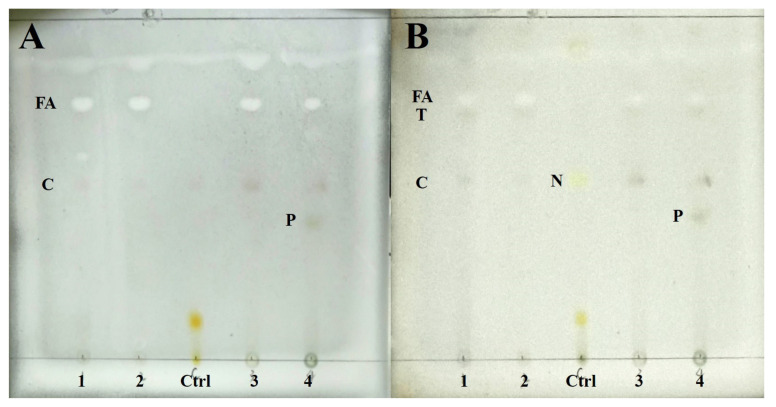
The result of TLC. (**A**) Wetting for color development; (**B**) Heating for color development. (1: *Peltula crispatula*; 2: *Peltula helanense*; 3: *Peltula reticulata*; 4: *Peltula overlappine*; Ctrl: *Lethariella cladonioides*. FA: fatty acid; T: terpenoid; C: cyathomorpha-unknown; N: norstictic acid; P: yellow-green pigment).

Chemical substances of four species were detected by TLC. The results show four species containing cyathomorpha-unknown, an unknown fatty acid (Rf = 48), and a terpenoid (Rf = 46), which distinguished previous studies (Figure 2). *P. overlappine* also contains a yellow-green pigment (Rf = 25.5).

### 3.3. Taxonomy

#### 3.3.1. The New Species

**Figure 3:**
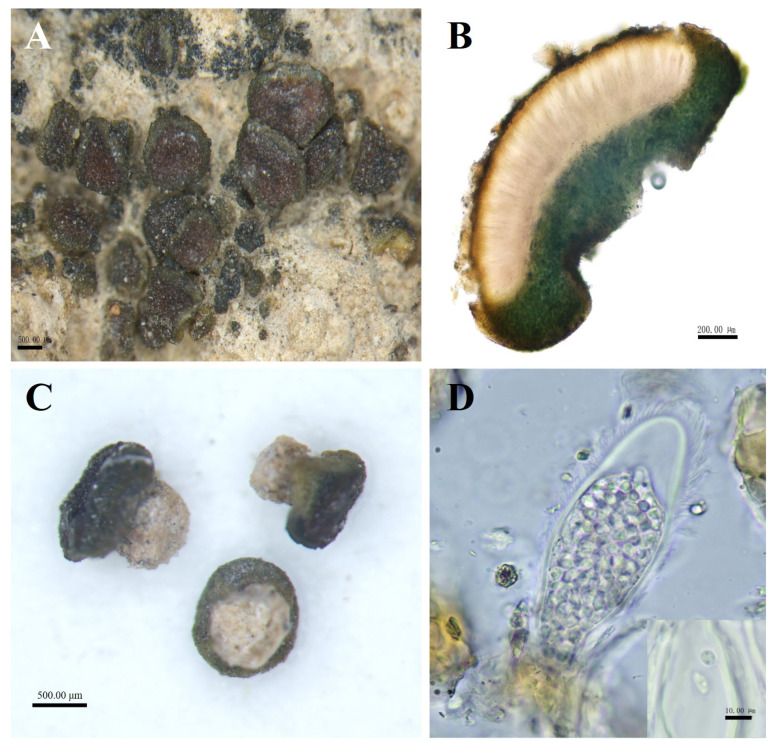
The morphological and anatomic structures of the thallus of *Peltula helanense*. (**A**) Squamulose thallus with apothecia; (**B**) Section of thallus; (**C**) Creamy-white cylindrical rhizoids; (**D**) Ascus and ascospores. Bars: A = 500.00 μm; B = 200.00 μm; C = 500.00 μm; D = 10.00 μm.

*Peltula helanense* S.Y. Wang and D.L. Niu, sp. nov. (Figure 3).

Fungal Names No.: FN 572013.

Etymology: First found on rocks in Helan Mountain.

Type: China, Ningxia Prov, Helan Mountain, Maliankou Gangouliang, 1616.1 m elev., on rock, 38°32′20″ N, 105°55′16.5″ E, 6 October 2014, D.L. Niu, S.L. Ma, T.T. Wang, and J.Q. Chen. DNA voucher: 14071046; GenBank Accession nos: PP468347 (ITS), PP471252 (LSU).

Diagnosis: Tiny, up to only 1 mm, creamy-white cylindrical rhizoids, with apothecia of the mature occupying almost the entire upper surface of thallus. The side of thallus with umbilicus looks like an open umbrella.

Description: Thallus squamulose, olive-brownish, 0.1–1 mm in diameter, rounded to subrounded, flat or raised. Upper surface lobed, margins entire, curled downwards and darker, not pruinose. Scattered individually, cemented to the substrate by creamy-white cylindrical rhizoids, the area usually occupies two-thirds of the lower surface. Thallus 479–794 μm thick, homoemerous. Upper cortex composed of 2–4 layers of dead algal cells, yellowish brown, 15–28 μm thick; lower cortex 33–58 μm thick, made up of yellowish rounded cells; algal layer encircled, 185–347 µm thick; medulla with rare hyphae. Apothecia red, immersed, round to subround, flat or slightly concave, 1 per squamule. Mature apothecia diameter of up to 0.8 mm, grow to occupy almost the entire upper surface of squamule. Hymenium 294–447 μm thick, K−, I+ wine red; epihymenium yellow-brown, 23–58 μm thick, K−, I+ wine red; subhymenium, transparent and colorless, 35–47 μm thick, IKI+ blue after K pretreatment. Asci clavate, with lacerated gelatinous sheath, 85–137 × 27–48 μm, more than 60 spored; ascospores simple, colorless, spherical or ellipsoidal, 2–5 × 2.5–5 μm; paraphyses segregated, apically slightly expanded, apical mycelial cells 5–7 μm thick, middle mycelial cells 3.5–4.5 μm thick. Pycnidia not seen; no soredia.

Ecology and distribution: *P. helanense* is harvested from Helan Mountain in China. It grows on rocks and occurs only in China.

Chemistry: K-, C-, KC-, I-, KI-. An unknown fatty acid, an unknown terpenoid, and cyathomorpha-unknown.

Notes: *P. helanense* is morphologically very similar to *P. obscurans var. deserticola* in that both thallus are small, squamulose, rounded or suborbicular, and have red and immersed apothecia. But *P. helanense* has only about 64 ascospores, whereas *P. obscurans var. deserticola* has more than 100. *P. obscurans var. deserticola* adheres to the substrate mainly by rhizines and umbilicus [5,6], while *P. helanense* is attached to the substrate by creamy-white cylindrical rhizoids.

Additional specimens examined: China, Ningxia Prov, Helan Mountain, Maliankou, 1682.5 m elev., on rock, 38°32′8.8″ N, 105°54′27″ E, 21 September 2014, D.L. Niu, S.L. Ma, F. Chen, and G.M. Zhang. DNA voucher: 14020467.

**Figure 4:**
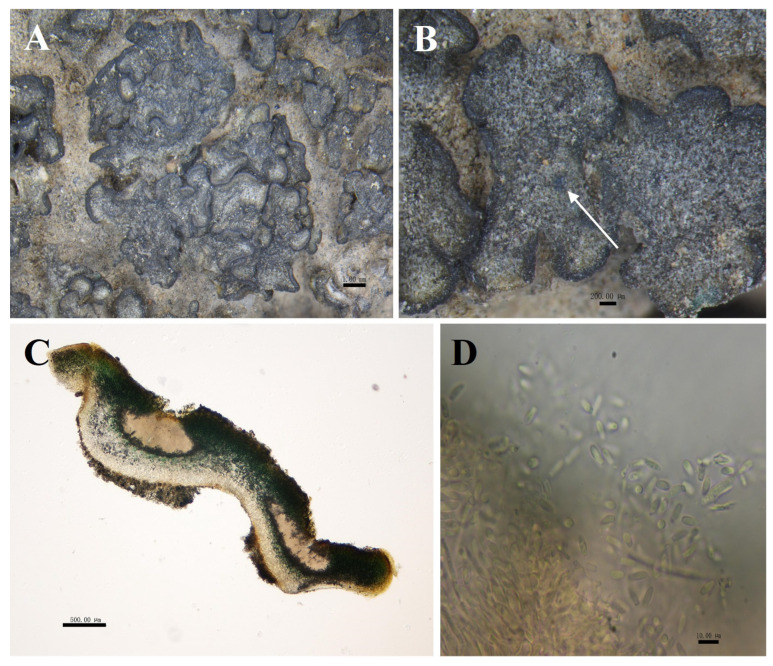
The morphological and anatomic structures of the thallus of *Peltula overlappine*. (**A**) Superimposed thallus; (**B**) Mature pycnidia (indicated by the arrow); (**C**) Section of thallus (showing pycnidia); (**D**) Conidia. Bars: A = 1.00 mm; B = 200.00 μm; C = 500.00 μm; D = 10.00 μm.

*Peltula overlappine* S.Y. Wang and D.L. Niu, sp. nov. (Figure 4).

Fungal Names No.: FN 572014.

Etymology: Squamules are closely stacked or clustered to form a thick, large patch.

Type: China, Ningxia Prov, Helan Mountain, suyukou, 1285 m elev., on soil, 38°41′47.1″ N, 106°00′3.4″ E, 12 July 2022, D.L. Niu, G.G. Zhao, Z.Q. Li and Y. Yang. DNA voucher: 22071279; GenBank Accession nos: PP468350 (ITS), PP471255 (LSU).

Diagnosis: Foliaceous squamules, distinctive thickened margins, mostly closely stacked or clustered.

Description: Thallus squamulose, grey-green to olive-green, about 1–3 mm in diameter. Rounded or irregularly shaped, flat to concave deeply. Margins black, thickened, often wavy or shallowly undulating, 75 μm wide. Upper surface usually has several shallow cracks, and the lower surface caramel-colored, attached to the substrate by a tuft of rhizoids, hyphae pale brown. Squamulose closely stacked or clustered, 6.5–10 × 5–7.1 cm, occasionally scattered individually. Thallus 340–758 μm thick, heteromerous. Upper cortex not developed, with a yellowish epinecral layer, made up of yellowish rounded cells, 39–79 μm thick; photobiont layer 235–353 µm thick; medulla with loosely distributed hyphae, 150–205 μm thick; lower cortex of 3–6 layers of hyaline spherical cells, 87–100 μm thick. Pycnidia cerebral, immersed, 529–647 × 235–647 μm, often 1–4 per, mature pycnidia appearing as black warty dots on the surface of squamulose; conidia ellipsoidal or clavate, 3.5–5 × 2–3.5 μm. Ascocarp not seen; no soredia.

Ecology and distribution: *P. overlappine* is harvested from Helan Mountain in China. It grows on sandy soils and occurs only in China.

Chemistry: K−, C−, KC−, I−, KI−. An unknown fatty acid, an unknown terpenoid, cyathomorpha-unknown, and a yellow-green pigment.

Notes: *P. overlappine* is morphologically similar to *P. patellata* and *P. subpatellata*. Moreover, it is phylogenetically highly correlated with *P. patellata*. All three species are squamulose, grey-green to olive-green, rounded to irregularly shaped. However, margins of *P. overlappine* are thickened up to 75 μm wide, usually wavy or shallowly undulating, with superimposed or clustered scale leaves, distinguishing it from *P. subpatellata* and *P. patellata*. The surface of *P. subpatellata* usually has yellow pruinose [11], while *P. overlappine* has no pruinose. *P. overlappine* are all negative, and TLC measured cyathomorpha-unknown, an unknown fatty acid, a terpenoid, and a yellow-green pigment. No substances were detected for *P. patellata* and *P. subpatellata* [6,11]. Conidia of *P. overlappine* are ellipsoidal or clavate, 3.5–5×2–3.5 μm, larger than those of *P. patellata* and *P. subpatellata*.

**Figure 5:**
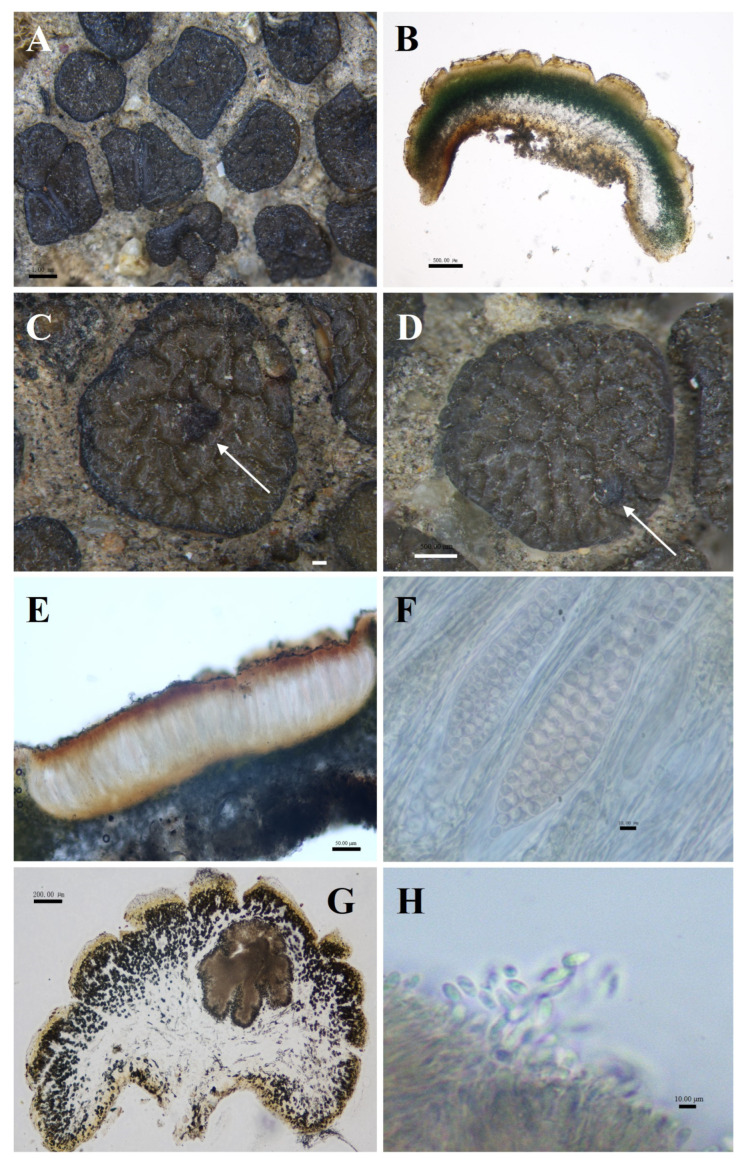
The morphological and anatomic structures of the thallus of *Peltula reticulata*. (**A**) Squamulose thallus; (**B**) Section of thallus; (**C**) Apothecia (indicated by the arrow); (**D**) Mature pycnidia (indicated by the arrow); (**E**) Section of apothecia; (**F**) Ascus and ascospores; (**G**) Section of pycnidia; (**H**) Conidia. Bars: (**A**) = 1.00 mm; (**B**) = 500.00 μm; (**C**) = 200.00 μm; (**D**) = 500.00 μm; (**E**) = 50.00 μm; (**F**) = 10.00 μm; (**G**) = 200.00 μm; (**H**) = 10.00 μm.

*Peltula reticulata* S.Y. Wang and D.L. Niu, sp. nov. (Figure 5).

Fungal Names No.: FN 572015.

Etymology: Brownish brown squamulose, upper surface densely reticulate-parted.

Type: China, Ningxia Prov, Helan Mountain, suyukou, 1292 m elev., on soil, 38°41′45.7″ N, 105°59′58.9″ E, 12 July 2022, D.L. Niu, G.G. Zhao, Z.Q. Li, and Y. Yang. DNA voucher: 22071277; GenBank Accession nos: PP468349 (ITS), PP471254 (LSU).

Diagnosis: Squamulose is densely reticulated with deep and uniform cracks.

Description: Thallus squamulose, brownish brown, 1–4 mm in diameter, suborbicular or irregularly shaped. Upper surface densely reticulate-parted; lower surface light brown to caramel brown, scattered individually, attached to the substrate by dense rhizoids, hyphae light brown; margin entire, deepened in color, occasionally bluntly serrate. Thallus 617–1353 μm thick, heteromerous, transparent membranous layer 35–58 μm thick in the outermost. Upper cortex of 3–6 layers of dead algal cells, yellowish brown, 48–120 μm thick; photobiont layer 88–235 μm thick; medulla mainly of loosely distributed hyphae, 235–623 μm; lower cortex of yellowish rounded cells, 88–235 μm thick. Pycnidia cerebral, immersed, 157–647 × 382–647 μm, often 1–2 per, mature pycnidia appearing as black warty dots on the surface of squamulose; conidia ellipsoidal or clavate, 1.5–4.5 × 2.5–5 μm. Apothecia reddish brown, immersed, round to subround, flat or slightly concave, 1 per squamule, 277–615 μm in diameter. Hymenium 451.5–554 μm thick, K−, I+ wine red; epihymenium brown, 59–82 μm thick, K−, I+ wine red; subhymenium, transparent and light brown, 6.5–13 μm thick, K−, I−. Asci clavate, with lacerated gelatinous sheath, 164–228.5 × 40–57 μm, more than 100 spored; ascospores simple, colorless, spherical, 5–8.5 × 5–8.5 μm; paraphyses segregated, apically slightly expanded, apical mycelial cells 2–4 μm thick, middle mycelial cells 1.5–3 μm thick. No soredia.

Ecology and distribution: *Peltula reticulata* is harvested from Helan Mountain in China. It grows on sandy soils and occurs only in China.

Chemistry: K−, C−, KC+ red-orange, I−, KI−. An unknown fatty acid, an unknown terpenoid, and cyathomorpha-unknown.

Notes: *P. reticulata* is closely related to *P. polyspora* in the phylogenetic tree, but the two species are morphologically distinct. *P. reticulata* is brownish, reticulately lobed from juvenile to mature, bluntly serrated on the underside of the margins, no pruinose, and with a 20–30 μm hyaline lamina in the outermost layer. In contrast, *P. polyspora* is olive-greenish brown, with smooth, entire margins, rarely lobed, yellowish pruinose, and without a hyaline layer. And *P. reticulata* has more spores than *P. polyspora* [11]. In contrast to the dense, deep fissures of mature *P. impressula*, which are more profound, the more mature and more significant the individual, juveniles have no fissures. *P. reticulata* is uniformly fissured and cracked from juvenile to mature.

Additional specimens examined: China, Ningxia Prov, Helan Mountain, Xiaoshuigou, 1180.3 m elev., on soil, 38°54′2.8″ N, 106°12′43.1″ E, 25 October 2014, D.L. Niu, S.L. Ma, J.M. Ma, and F. Chen. DNA voucher: 14021449; China, Ningxia Prov, Helan Mountain, Dashuigou, 1205 m elev., on soil, 38°51′11.5″ N, 106°10′56.8″ E, 22 September 2017, D.L. Niu. DNA voucher: 170743.

#### 3.3.2. The New Records

**Figure 6:**
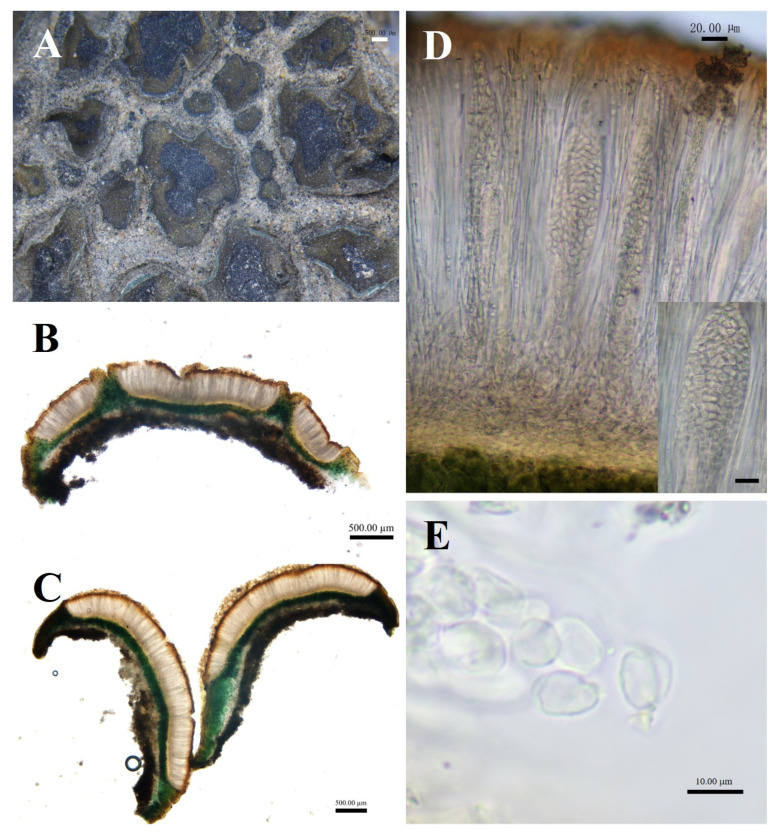
The morphological and anatomic structures of the thallus of of *Peltula crispatula*. (**A**) Squamulose thallus; (**B**,**C**) Section of thallus; (**D**) Hymenium and ascus; (**E**) Ascospores; Bars: (**A** = 1.00 mm; (**B**,**C**) = 500.00 μm; (**D**) = 20.00 μm; (**E**) = 10.00 μm.

*Peltula crispatula* (Nyl.) Egea, Biblthca Lichenol. 31:64 (1989) (Figure 6).

Specimens examined: China, Ningxia Prov, Helan Mountain, Daolugou, 1282.1 m elev., on soil, 39°16′56.9″ N, 106°39′45.6″ E, 1 November 2014, D.L. Niu, J.M. Ma, G.M. Zhang, and F. Pu. DNA voucher: 14011623; GenBank Accession nos: PP468345 (ITS), PP471250 (LSU).

Diagnosis: Squamulose is irregularly shaped and attached to the substrate’s surface by dense hyphae.

Description: Thallus squamulose, olive-green, juvenile squamulose suborbicular, mature squamulose irregularly shaped, 0.5–3 mm in diameter. Margins slightly darker in color, entire or lobed, flat or curled upwards on all sides; lower surface brownish, attached to the substrate surface with dense rhizoids, scattered individually. Thallus 388–623.5 μm thick, heteromerous. Upper cortex not developed, a yellowish membranous layer of 35–58 μm thick; photobiont layer 200–412 μm thick, upper layer of 2–4 layers of dead yellow algal cells, yellow-brown, 61.5–102.5 μm thick; lower layer of living algae, 88–309.5 μm thick; medulla closely spaced, 40–88 μm thick; lower cortex of 3–6 layers of yellow-brown globular cells, 17.5–47 μm thick. Apothecia reddish brown, immersed, flat or slightly concave, disc gradually expands with the growth of squamule, initially rounded, then becoming irregular with the shape of squamule, 0.2–2 mm in diameter. Hymenium approximately 317.5–441 μm thick, K-, I+ wine red; epihymenium yellow-brown, 41–76.5 μm thick, K−, I+ wine red; subhymenium, transparent and light yellow, 23.5–41 μm thick, IKI+ blue after K pretreatment. Ascus clavate, with lacerated gelatinous sheath, 200–250 × 25–40 μm, spores over 100; ascospores simple, colorless, spherical or ellipsoidal, 3–7 × 3–5.5 μm; paraphyses segregated, apically slightly expanded, apical mycelial cells 3.5–5.5 μm thick, middle mycelial cells 1.5–3.5 μm thick. Pycnidia not seen; no soredia.

Ecology and distribution: *P. crispatula* is harvested from Helan Mountain in China. It grows on sandy soils in N Sahara, Tunisia, Algeria, and S Spain; the first occurrence in China.

Chemistry: K−, C−, KC−, I−, KI−. An unknown fatty acid, an unknown terpenoid, and cyathomorpha-unknown.

Notes: *P. crispatula* is morphologically similar to *P. psammophila*. Both have scale-like olive-green squamuloses that are rounded to irregularly shaped. However, whereas *P. psammophila* adheres to the substrate in bundles of hyphae, apothecia up to 1 mm [6]; *P. crispatula* adheres to the substrate in very dense hyphae, apothecia can reach up to 2 mm.

Additional specimens examined: China, Ningxia Prov, Helan Mountain, Daolugou, 1177.2 m elev., on soil, 39°17′06″ N, 106°39′42″ E, 30 April 2016, D.L. Niu. DNA voucher: 160047.

### 3.4. Key to Peltula Species from Helan Mountain

1. Thallus on rock⋯⋯⋯⋯⋯⋯⋯⋯⋯⋯⋯⋯⋯⋯⋯⋯⋯⋯⋯⋯⋯⋯⋯⋯⋯⋯⋯⋯⋯2.
1. Thallus on soil⋯⋯⋯⋯⋯⋯⋯⋯⋯⋯⋯⋯⋯⋯⋯⋯⋯⋯⋯⋯⋯⋯⋯⋯⋯⋯⋯⋯⋯5.
2. Thallus squamules⋯⋯⋯⋯⋯⋯⋯⋯⋯⋯⋯⋯⋯⋯⋯⋯⋯⋯⋯⋯⋯⋯⋯⋯⋯⋯⋯3.
2. Thallus peltate⋯⋯⋯⋯⋯⋯⋯⋯⋯⋯⋯⋯⋯⋯⋯⋯⋯⋯⋯⋯⋯⋯⋯⋯⋯⋯⋯⋯⋯4.
3. Squamules brownish-yellow or olive-colored, black soredia on top
⋯⋯⋯⋯⋯⋯⋯⋯⋯⋯⋯⋯⋯⋯⋯⋯⋯⋯⋯⋯⋯⋯⋯⋯⋯⋯⋯⋯⋯⋯⋯⋯⋯⋯⋯⋯⋯⋯*P. impressa.*
3. Squamules olive-brownish, mature apothecia covered the whole upper surface
⋯⋯⋯⋯⋯⋯⋯⋯⋯⋯⋯⋯⋯⋯⋯⋯⋯⋯⋯⋯⋯⋯⋯⋯⋯⋯⋯⋯⋯⋯⋯⋯⋯⋯⋯⋯⋯⋯*P. helanense.*
4. Flat or undulate, up to 15 mm; margin dark grey-black, ascocarp buried, black warty-punctate, 1–5 per scale⋯⋯⋯⋯⋯⋯⋯⋯⋯⋯⋯⋯⋯⋯⋯⋯⋯⋯⋯⋯⋯⋯⋯⋯⋯⋯*P. aricana.*
4. Not undulate, up to 12 mm in diameter, sometimes with lip-shaped mound of black soredia at the margin⋯⋯⋯⋯⋯⋯⋯⋯⋯⋯⋯⋯⋯⋯⋯⋯⋯⋯⋯⋯⋯⋯⋯⋯⋯⋯⋯⋯⋯⋯⋯*P. euploca.*
5. Thallus brownish, upper surface densely reticulate-parted, blunt serrated edges visible on margins⋯⋯⋯⋯⋯⋯⋯⋯⋯⋯⋯⋯⋯⋯⋯⋯⋯⋯⋯⋯⋯⋯⋯⋯⋯⋯⋯⋯⋯⋯⋯⋯⋯⋯*P. reticulata.*
5. Thallus olive-colored, upper surface lobed or smooth⋯⋯⋯⋯⋯⋯⋯⋯⋯6.
6. Upper surface with small depressions resembling cyphellae, underdeveloped epithelial layer, translucent⋯⋯⋯⋯⋯⋯⋯⋯⋯⋯⋯⋯⋯⋯⋯⋯⋯⋯⋯⋯⋯⋯⋯⋯⋯⋯⋯⋯⋯*P. impressula.*
6. Upper cortex 39–79 μm thick with a layer of yellow dead algal cells⋯7.
7. Surface usually yellow pruinose⋯⋯⋯⋯⋯⋯⋯⋯⋯⋯⋯⋯⋯⋯⋯⋯⋯⋯⋯⋯*P. subpatellata.*
7. No pruinose on the surface⋯⋯⋯⋯⋯⋯⋯⋯⋯⋯⋯⋯⋯⋯⋯⋯⋯⋯⋯⋯⋯⋯8.
8. Squamules foliated, distinctive thickened margins, mostly closely clustered or overlapping⋯⋯⋯⋯⋯⋯⋯⋯⋯⋯⋯⋯⋯⋯⋯⋯⋯⋯⋯⋯⋯⋯⋯⋯⋯⋯⋯⋯⋯⋯⋯*P. overlappine.*
8. Squamules, scattered individually⋯⋯⋯⋯⋯⋯⋯⋯⋯⋯⋯⋯⋯⋯⋯⋯⋯⋯⋯9.
9. Hymenium with ample oil droplets⋯⋯⋯⋯⋯⋯⋯⋯⋯⋯⋯⋯⋯⋯⋯⋯⋯⋯*P. radicata.*
9. Hymenium without oil droplets⋯⋯⋯⋯⋯⋯⋯⋯⋯⋯⋯⋯⋯⋯⋯⋯⋯⋯⋯⋯10.
10. Containing three secondary metabolites⋯⋯⋯⋯⋯⋯⋯⋯⋯⋯⋯⋯⋯⋯⋯*P. crispatula.*
10. No substances detected by TLC⋯⋯⋯⋯⋯⋯⋯⋯⋯⋯⋯⋯⋯⋯⋯⋯⋯⋯⋯*P. richardsii.*

## 4. Discussion

Four species of *Peltula* growing in Helan Mountain were described. The genetic relationships between species of *Peltula* were revealed in phylogenetic analyses using two loci as markers. DNA sequences can explain phylogenetic relationships for species that are difficult to define by relying on morphology. *P. overlappine*, *P. patellata*, *P. reticulata*, and *P. polyspora* all show high posterior probabilities in the phylogenetic tree but are morphologically quite different. This study concludes that morphological observations must be combined with molecular data to identify lichen species more accurately.

Helan Mountain has been seriously neglected in lichen research. In addition to the four species recorded in the text, we found seven other reported species of *Peltula*. This suggests that Helan Mountain lichens are rich in species and a hotspot for lichen distribution. Among them, three species (*P. africana*, *P. impressa*, *P. helanense*) were found on rocks, while the remaining eight species (*P. crispatula*, *P. euploca*, *P. impressula*, *P. overlappine*, *P. radicata*, *P. reticulata*, *P. richardsii*, *P. subpatellata*) grew on the soil surface and formed biological soil crusts. It is assumed that this is related to the geographic environment of Helan Mountain, where most of the species in this study were collected from the premontane desert steppe area, where the arid climate with little rainfall, sparse vegetation, and calcium-rich soil provides a favorable environment for the growth of lichens of *Peltula*.

## 5. Conclusions

In this study, three new species (*Peltula helanense* S.Y. Wang and D.L. Niu, *P. overlappine* S.Y. Wang and D.L. Niu, and *P. reticulata* S.Y. Wang and D.L. Niu) and a new record species (*P. crispatula* (Nyl.) from China are described from Helan Mountain in northwest China. At present, we have identified a total of 11 species (*P. africana*, *P. crispatula*, *P. euploca*, *P. impressa*, *P. impressula*, *P. helanense*, *P. overlappine*, *P. radicata*, *P. reticulata*, *P. richardsii*, *P. subpatellata*) of *Peltula* distributed in Helan Mountain. This suggests that Helan Mountain is by far the richest area in China in terms of lichen species diversity in *Peltula*. Based on this study, the importance of lichen research in Helan Mountain is also demonstrated.

## 6. Future Prospectives

We are continuing to delve deeper, with some specimens yet to be identified. We will continue to study the species composition and ecological distribution of *Peltula* in the desert steppe area and which species dominate biological soil crusts.

## Figures and Tables

**Table 1:** Specimen and sequence information used for molecular phylogenetic analysis.

Species Names	Voucher Specimen	GenBank Accession Numbers	
		LSU	ITS
*Peltula africana*	1990, South Africa, 14304b	MF766384	MF766343
*P. africana*	2010, China, 100223	**PP471343**	MN103150
*P. anthracina*	2010, Brazil, CGMS 385	-	MW267988
*P. anthracina*	2010, Brazil, CGMS 384	-	MW267989
*P. auriculata*	1992, Venezuela, 24901	DQ832330	DQ832329
*P. auriculata*	1992, Venezuela, 24902	MF766385	MF766344
*P. bolanderi*	1993, Mexico, 20196e	MF766386	MF766345
*P. boletiformis*	2003, South Africa, 14911a-1	MF766387	MF766346
*P. capensis*	1994, South Africa, 14382b-2	MF766388	MF766347
*P. clavata*	1987, Australia, 18047a	MF766389	MF766348
*P. confusa*	2019, China, HMAS-L 154718	OP429694	OP429728
*P. confusa*	2019, China, HMAS-L 154719	OP429695	OP429729
*P. congregata*	2003, South Africa, 14909b-1	MF766390	MF766349
*P. coriacea*	2003, South Africa, 14500a-1	MF766391	MF766350
*P. corticola*	2002, Yemen, 14201	MF766419	MF766378
*P. crispatula*	1987, Morocco, 21001a	MF766392	MF766351
*P. crispatula*	2014, China, 14011623	PP471250	PP468345
*P. cylindrica*	2003, South Africa, 14920a-1	MF766393	MF766352
*P. euploca*	1993, Mexico, 20162a	MF766394	MF766353
*P. farinosa*	1993, Mexico, 20119a	MF766396	MF766355
*P. hassei*	1994, South Africa, 14354a	MF766406	MF766365
*P. helanense*	2014, China, 14020467	**PP471251**	**PP468346**
*P. helanense*	2014, China, 14071046	**PP471252**	**PP468347**
*P. impressa*	1993, Mexico, 20140f	MF766398	MF766357
*P. impressa*	2014, China, 14021492	**PP471344**	OR610321
*P. impressa*	2022, China, 22071276	**PP471345**	OR610322
*P. impressula*	2017, China, HMAS-L 154744	OP429701	OP429740
*P. impressula*	2017, China, HMAS-L 154745	OP429702	OP429741
*P. inversa*	2001, Namibia, 15058	MF766399	MF766358
*P. leptophylla*	1993, Mexico, 20128a	MF766400	MF766359
*P. lingulata*	1994, South Africa, 14452a	MF766401	MF766360
*P. lobulata*	2019, China, HMAS-L 145468	MT499313	MT499291
*P. lobulata*	2019, China, HMAS-L 145469	MT499314	MT499292
*P. marginata*	2003, South Africa, 14920d-1	MF766402	MF766361
*P. michoacanensis*	1993, Mexico, 20140l	MF766403	MF766362
*P. obscuratula*	2019, China, HMAS-L 154748	OP429707	OP429734
*P. obscuratula*	2019, China, HMAS-L 154749	OP429709	OP429736
*P. obscurans var. deserticola*	2003, South Africa, 14900b-1	MF766404	MF766363
*P. obscurans var. deserticola*	2003, South Africa, 14902d-1	MF766405	MF766364
*P. omphaliza*	1993, Mexico, 20148b	MF766408	MF766367
*P. overlappine*	2022, China, 22071279	**PP471255**	**PP468350**
*P. patellata*	2003, Mexico, 16254b	MF766409	MF766368
*P. placodizans*	1993, Mexico, 20112a	MF766410	MF766369
*P. polycarpa*	2019, China, HMAS-L 145471	MT499319	MT499300
*P. polycarpa*	2019, China, HMAS-L 145472	MT499320	MT499301
*P. polyphylla*	2019, China, HMAS-L 145475	MT499326	MT499303
*P. polyphylla*	2019, China, HMAS-L 145474	MT499325	MT499304
*P. polyspora*	2019, China, HMAS-L 154721	OP429706	OP429732
*P. polyspora*	2019, China, HMAS-L 154723	OP429704	OP429733
*P. psammophila*	2018, Germany, BM 761074	MF766411	MF766370
*P. pseudoboletiformis*	2019, China, HMAS-L 145476	MT499322	MT499297
*P. pseudoboletiformis*	2019, China, HMAS-L 145477	MT499324	MT499299
*P. radicata*	2002, Yemen, 14241a	MF766412	MF766371
*P. reticulata*	2017, China, 170743	**PP471253**	**PP468348**
*P. reticulata*	2022, China, 22071277	**PP471254**	**PP468349**
*P. richardsii*	1993, Mexico, 20194a	MF766413	MF766372
*P. rodriguesii*	1990, Namibia, 15901	MF766414	MF766373
*P. santessonii*	2003, South Africa, 14912b-1	MF766415	MF766374
*P. sonorensis*	1993, Mexico, 20196d	MF766416	MF766375
*P. submarginata*	2019, China, HMAS-L 145480	MT499316	MT499294
*P. submarginata*	2019, China, HMAS-L 145481	MT499318	MT499295
*P. subpatellata*	2019, China, HMAS-L 154732	OP429681	OP429714
*P. subpatellata*	2019, China, HMAS-L 154733	OP429682	OP429715
*P. tortuosa*	1996, Venezuela, 24039b	MF766417	MF766376
*P. umbilicata*	2003, South Africa, 14901a-1	DQ832334	DQ832333
*P. zahlbruckneri*	1993, Mexico, 20157a	MF766418	MF766377
*Peccania terricola*	2018, China, 201899118	OM523033	OM523029
*Peccania* sp.	2019, China, HMAS-L 154764	OP429711	OP429744
*Peccania* sp.	2019, China, HMAS-L 154765	-	OP429745

Note: The sequences generated in this study are represented in bold.

## Data Availability

The names of the new species were formally registered in the Fungal Names database (https://nmdc.cn/fungalnames, accessed on 24 June 2024). Specimens were deposited in Ningxia Agricultural College (NXAC), Ningxia University (NXU). The newly generated sequences were deposited in GenBank (https://www.ncbi.nlm.nih.gov/genbank, accessed on 30 March 2024).
